# Claws and canines: injury patterns following European brown bear attacks

**DOI:** 10.1007/s12024-025-01001-y

**Published:** 2025-04-10

**Authors:** Richard Sivulič, Martin Janík, Veronika Rybárová, Filip Babiak, Ľubomír Straka

**Affiliations:** 1 Department of Forensic Medicine and Medicolegal Expertise, Jessenius Faculty of Medicine in Martin, Comenius University Bratislava, Martin, Slovakia; 2 Health Care Surveillance Authority, Bratislava, Slovakia

**Keywords:** Autopsy, Brown bear, Bite marks, Injury patterns, Wildlife

## Abstract

In recent years, bear attacks in Slovakia have increased, including two fatal attacks. The first fatality involved a 63-year-old man who was attacked by a brown bear while hiking with his family. He sustained grievous injuries to the left thigh and died at the scene shortly after the attack. In the second case, a 58-year-old man was found dead near a walking trail, and recent bear prints were found nearby. The man sustained various blunt and sharp injuries to the head and right upper extremity, strongly suggesting a bear attack. The cause of death was severance of the cervical spinal cord. Both victims presented with similar topographical and patterned injuries, which were consistent with biting and clawing. Sets of similar penetrating wounds arranged in rectangular patterns were also found on both victims. Differentiating such injuries from homicidal or self-inflicted wounds is of pivotal medico-legal importance. This paper provides a detailed analysis, visualization and assessment of wound morphology following fatal bear attacks.

## Case 1

A 63-year-old man was attacked by a European brown bear while hiking in the mountains with family members. Despite resuscitative attempts, the man was pronounced dead shortly after the attack. A medico-legal autopsy of the body was performed.

**Fig. 1:**
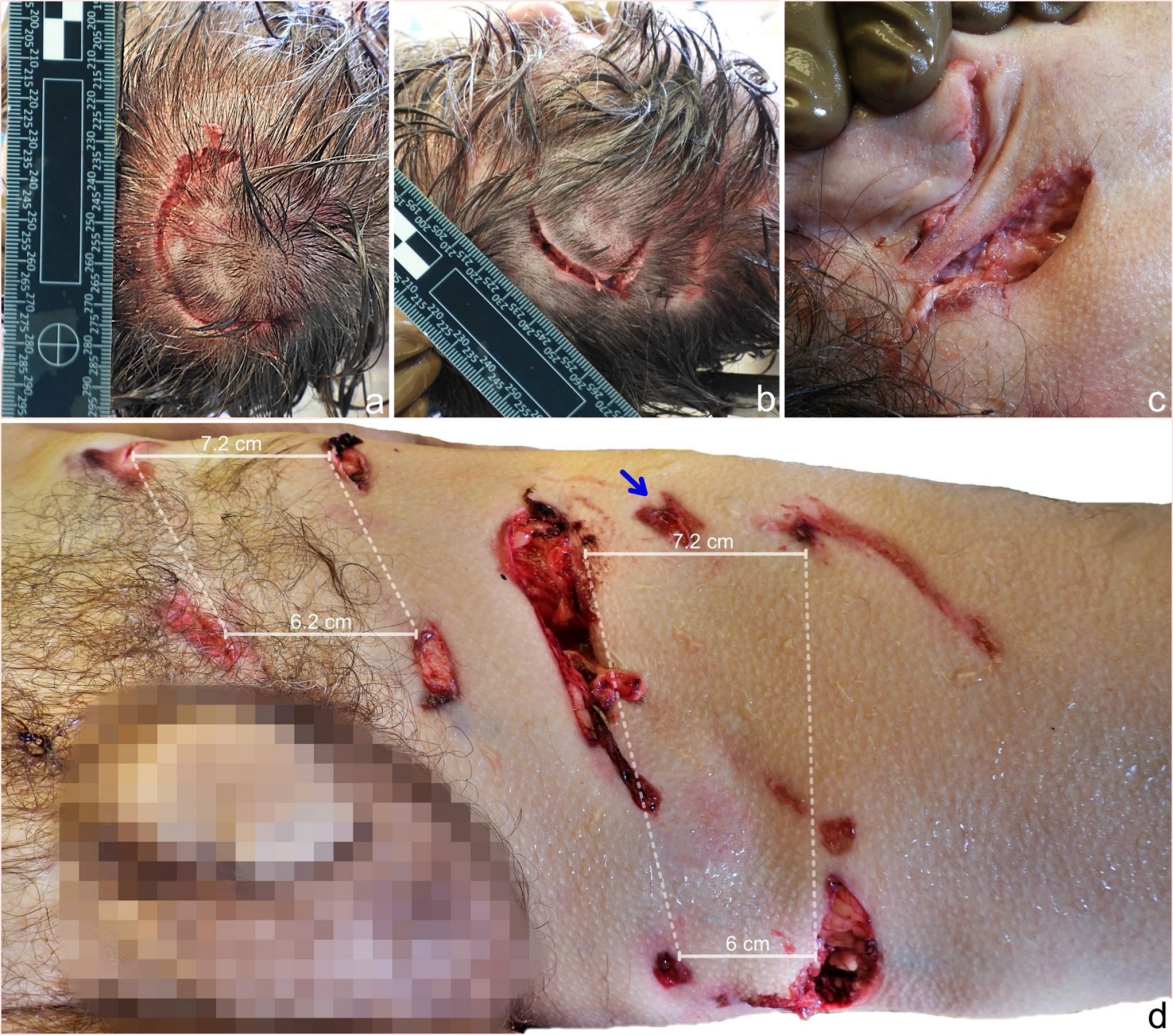
**a**– An arcuate laceration in the occipital area of the scalp, which was attributed to clawing **b**– A V-shaped laceration with scalp avulsion in the occipitoparietal area of the scalp, which was attributed to clawing **c**– A pair of lacerations below the right auricle, which were both attributed to clawing **d**– The anteromedial side of the left thigh and left groin with two sets of puncture wounds arranged in a rectangular pattern, which were attributed to biting. The laceration and abrasion between the upper pair of canines were likely caused by incisors (blue arrow). The large spacing between the wounds indicates that they were caused by maxillary canines, which is useful for determining the position of the bear during the attack

External examination revealed that the well-nourished man was dressed in bloodstained and damaged hiking attire. An arcuate laceration in the occipital area of the scalp measuring 6 cm in length was found (Fig. 1a). In addition, a V-shaped laceration with scalp avulsion measuring 4 cm in length and 2 cm in width was found in the occipitoparietal area (Fig. 1b), as well as a pair of lacerations below the right auricle, each measuring 4 cm in length (Fig. 1c). Furthermore, numerous scratch-like abrasions were present on the forehead. Multiple puncture wounds arranged in rectangular patterns were found on the anteromedial side of the left thigh and the left groin. The morphologies of these wounds varied, presenting with spindle-shaped, triangular or circular shapes and irregular, abraded margins (Fig. 1d). The largest defect projected as a wound channel was in the thigh musculature and was connected with a wound diagonally below it. On the right forearm, puncture wounds with a similar rectangular pattern were noted. Internally, there was extensive damage to the left femoral musculature and complete transection of the left femoral artery and left femoral vein. The internal thoracic and abdominal organs appeared pale. The only natural disease condition was moderate atherosclerosis of the aorta. Toxicological analyses of blood and urine samples were negative. The cause of death was determined to be multiple injuries due to bear mauling.

## Case 2

The body of a 58-year-old man was found dead near a forest walking trail. A box cutter and a damaged cell phone were found beside the body. Additionally, recent bear prints were discovered near the scene. Signs of various blunt and sharp injuries to the head and the right upper extremities were found, strongly suggesting a bear attack. A medico-legal autopsy of the body was performed.

**Fig. 2:**
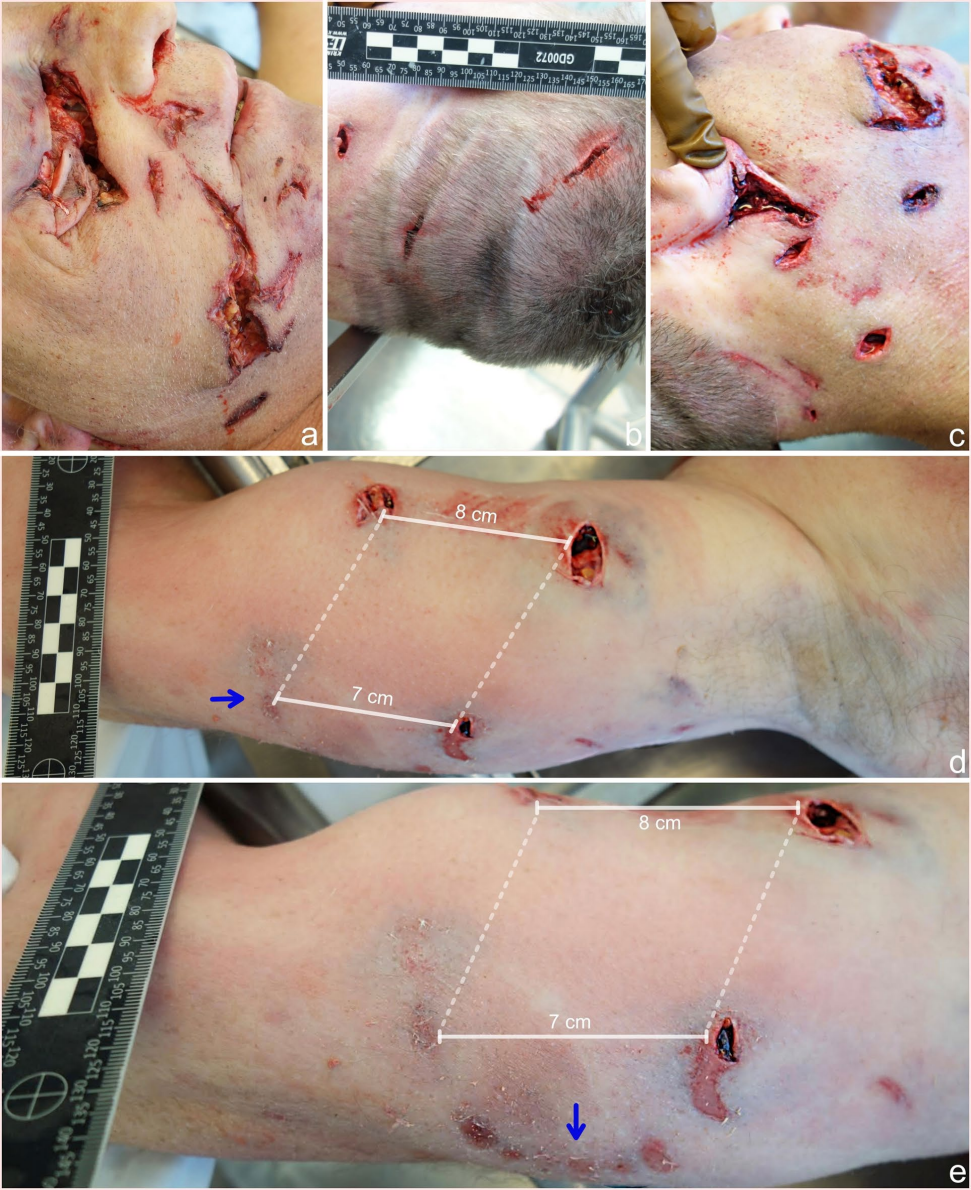
**a**– Lacerations on the right eye and inferior aspect of the right cheeks, which were attributed to biting **b**– Lacerations on the occipitoparietal area of the scalp, which were attributed to clawing **c**– A set of puncture wounds in the right mandibular region and deep lacerations below the right auricle, which were attributed to clawing **d**– A set of three puncture wounds on the right upper arm, which were attributed to biting. Note that the fourth injury on the bottom left side is superficial (blue arrow) **e**– Continuation of the rectangular injury pattern on the medial aspect of the right upper arm. Six superficial abrasions were found and were arranged in an arch-shaped pattern, matching the inferior incisors of a brown bear’s dentition (blue arrow) [18]

External examination revealed that the well-built man was dressed in torn and bloodstained outdoor clothing. Numerous irregular lacerations were found on the right side of the face, the largest of which was located over the right eye and the inferior part of the right cheek, measuring 6.5 × 4 cm and 8.5 × 2 cm in length and width, respectively (Fig. 2a). The right orbit and upper part of the right maxilla were fractured. Three lacerations were found on the occipitoparietal area of the scalp and nape, measuring from 1.0 to 3.3 cm in length (Fig. 2b). A set of three puncture wounds was found on the right mandibular angle, spaced 3 cm from each other (Fig. 2c). A deep laceration measuring 3.5 × 2.5 cm was found below the right auricle. Three puncture wounds in a rectangular pattern and one superficial abrasion surrounded by subcutaneous contusions were found on the upper right arm (Fig. 2d), and a set of six superficial abrasions arranged in an arch-shaped pattern was found on the mid-posterior aspect of the upper arm (Fig. 2e). Internal examination revealed rib fractures on the left side and a cervical spine fracture at the C5-C6 level with partial rupture and contusion of the spinal cord. In addition to the traumatic findings, the victim suffered from hypertensive heart disease and moderate to severe coronary atherosclerosis and had undergone bypass graft surgery. Toxicological analyses of blood and urine samples were negative. The cause of death was determined to be multiple injuries due to bear mauling.

## Discussion

**Fig. 3:**
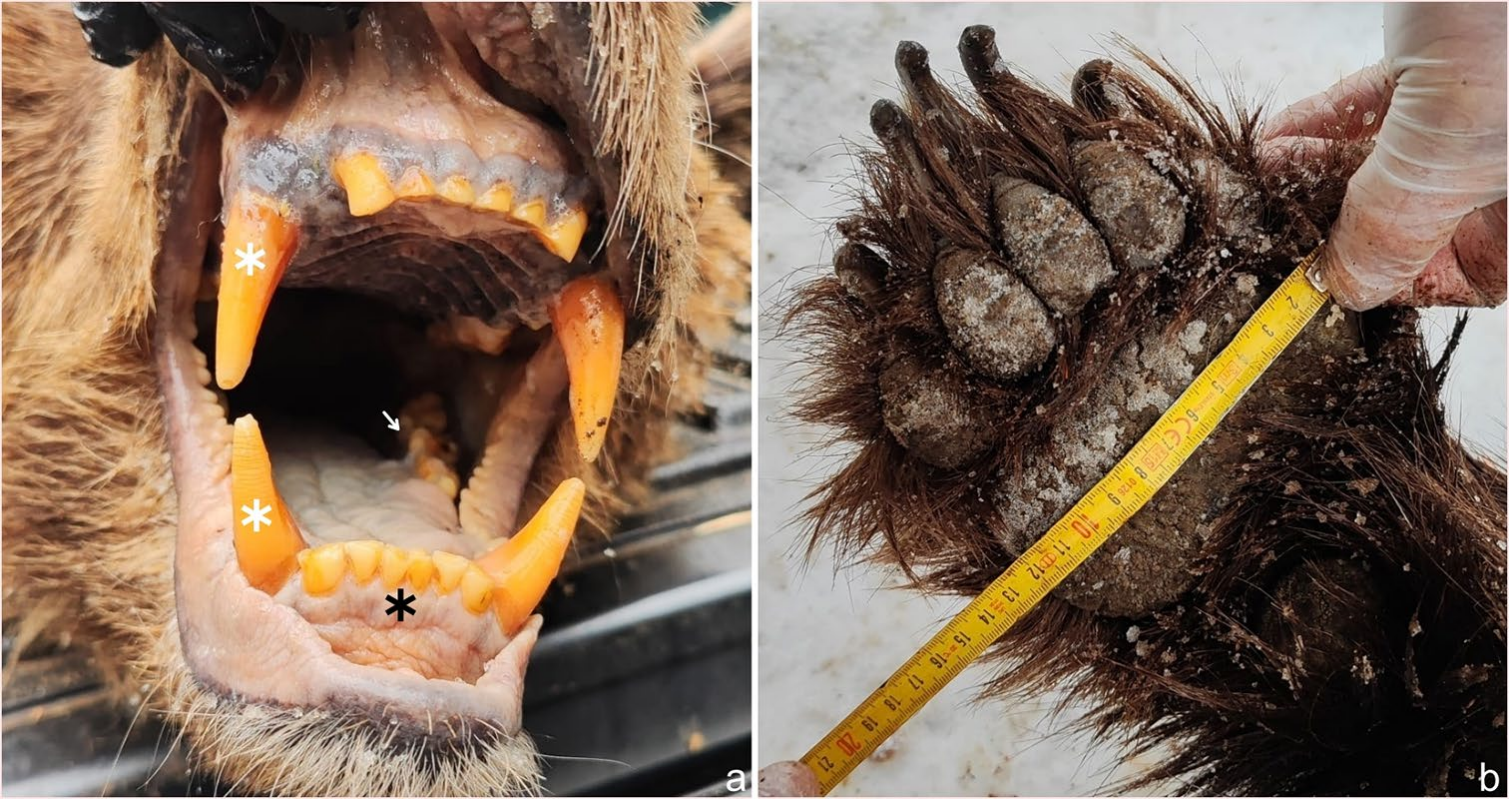
**a**– Dentition of a brown bear, consisting of six incisors (black asterisk) between each pair of canine teeth (white asterisk). Carnassial teeth are located behind the canine teeth (white arrow) **b**– Forefoot of a brown bear with five nonretractile claws

The European brown bear (*Ursus arctos arctos*) belongs to the extant family *Ursidae* and is characterized as a robust plantigrade carnivorans, with a body mass ranging from 150 to 250 kg [1, 2]. In addition to having a heavyset body profile, the dentition of the European brown bear consists of 42 teeth, including elongated tapering canines used for tearing and gripping, cuneiform incisors used for cutting, and carnassial teeth used for crushing and processing meat [2] (Fig. 3a). The forefeet (Fig. 3b) have slightly curved claws that are approximately twice as long as those on the hindfeet [3]. During attacks on humans, bears rear onto their hind legs and strike indiscriminately, with their forelimbs and teeth targeting the nearest body parts, primarily the neck, nape, and head regions [2, 4, 5]. The brown bear subsequently subdues its prey by holding it down with one forelimb while using the other forelimb to inflict injuries [5].

The appearance of a bite mark corresponds to the morphology of the teeth, their arrangement within the dental arch, the bite force applied and the movement of the jaws. The average length of the brown bear’s canines ranges between 3.4 and 4.3 cm and rarely exceeds 5.5 cm [6]. On the basis of the tapered ends, conical shape, and lateral placement of these canines within the jaw, the injury pattern consists of four circular puncture wounds in a rectangular arrangement, with the diameter of the punctures not exceeding 2.3 cm. The maxillary intercanine width is typically greater than the mandibular intercanine width, with the difference varying from a few millimeters to 1.5 cm [6]. The difference in the intercanine width reflected in the wounds also depends on the canine width and bite depth. The injury pattern formed by cuneiform incisors manifests as six adjacent abrasions among the canine wounds. The semi-arched arrangement of the maxillary incisors can be distinguished from the more linear arrangement of the mandibular incisors. In superficial bites, the diameter and depth of canine-inflicted wounds are reduced, and incisor marks may not be present [6]. Bite force is directly correlated with the severity of bite injuries, which primarily contribute to blunt trauma components, such as contusions and bone fractures. The average canine and molar bite forces of brown bears are 1627 N and 3175 N, respectively [7]. The force tolerance of adult facial bones (zygomatic bone and maxilla) ranges from 490 to 1800 N (50–183 kg), which are well within the bite force ranges of brown bears [8]. When ripping flesh from bones, the bear moves its jaws, resulting in torque, leading to the laceration and avulsion of superficial soft and deep tissues and altering the morphology of primary bite marks. In these two cases, the morphology of the puncture wounds on the left thigh differed due to the violent torque movement of the jaws, which led to ruptures of the underlying soft tissues, as shown in Fig. 1d. The limbs of brown bears include five nonretractile claws with lengths up to 8 cm, which are used for digging up tubers and small prey [3]. Injuries incurred by clawing and mauling range from superficial linear abrasions to avulsed lacerations with decollement [4]. Grievous injuries, such as neurocranial fractures and brain evisceration attributed to mauling, have also been described in the literature [9, 10].

Most large carnivore attacks in Europe involve brown bears [11]. The injuries caused by bear assaults predominantly occur in craniofacial regions or as defensive wounds on the upper extremities [4]. Dog attacks are associated with similar wound topographies, including soft tissue trauma; however, claw-inflicted injuries are absent [12]. The most frequent cause of death in bear attacks is exsanguination [5]. The less frequent causes include cervical spine injuries, brain evisceration and air embolism [5, 9, 10, 13]. In contrast, cervical trauma is the leading cause of death in attacks by large felids, such as lions, tigers, and leopards, due to their distinct hunting behavior [14–16]. The forensic relevance of deaths due to animal attacks lies in differentiating the observed injuries from homicidal and self-inflicted wounds [17]. The presented cases highlight the importance of meticulous external examination with morphometry of the injuries and site observation. Additionally, biological samples can be used to identify the animal responsible for the attack and improve investigative processes.
